# Lymphocyte activating gene 3 protein expression in nasopharyngeal carcinoma is correlated with programmed cell death-1 and programmed cell death ligand-1, tumor-infiltrating lymphocytes

**DOI:** 10.1186/s12935-021-02162-w

**Published:** 2021-08-28

**Authors:** Fan Luo, Jiaxin Cao, Feiteng Lu, Kangmei Zeng, Wenjuan Ma, Yan Huang, Li Zhang, Hongyun Zhao

**Affiliations:** 1 Department of Experimental Research, State Key Laboratory of Oncology in South China, Collaborative Innovation Center for Cancer Medicine, Sun Yat-Sen University Cancer Center, Guangzhou, China; 2 Department of Medical Oncology, State Key Laboratory of Oncology in South China, Collaborative Innovation Center for Cancer Medicine, Sun Yat-Sen University Cancer Center, 651 Dongfeng Road East, Guangzhou, 510060 Guangdong China; 3 Department of Intensive Care Unit, State Key Laboratory of Oncology in South China, Collaborative Innovation Center for Cancer Medicine, Sun Yat-Sen University Cancer Center, Guangzhou, China; 4 Department of Clinical Research, State Key Laboratory of Oncology in South China, Collaborative Innovation Center for Cancer Medicine, Sun Yat-Sen University Cancer Center, 651 Dongfeng Road East, Guangzhou, 510060 Guangdong China

**Keywords:** LAG-3, CD3, GZMB, PD-L1, PD-1, NPC

## Abstract

**Background:**

Immunotherapy has shown promising efficacy in patients with nasopharyngeal carcinoma (NPC). Lymphocyte activating 3 gene (LAG-3) represents a significant immune target, however, its relationship with NPC remains unclear. This study aimed to evaluate LAG-3 expression in NPC and its association with CD3+ tumor-infiltrating lymphocytes (TILs), Granzyme B (GZMB), programmed death ligand 1 (PD-L1), and programmed death 1 (PD-1) expression.

**Methods:**

A total of 182 patients with NPC from Sun Yat-sen University Cancer Center, China, were included in this retrospective study. LAG-3 expression in 15 NPC cell lines and LAG-3, CD3+ TILs, GZMB, PD-L1 and PD-1 in clinical samples were estimated using immunohistochemistry. The Chi-square test was used to estimate the association between LAG-3, other biomarkers, and clinical characteristics. Survival analysis was performed using the Kaplan–Meier method and the Cox regression model.

**Results:**

LAG-3 was negatively expressed in all of the 15 NPC cell lines, whereas, 147 patients with NPC (80.8%) exhibited high LAG-3 expression on TILs from tumor tissues. Male patients and those who were EBV-positive presented higher LAG-3 expression. Correlation analyses showed that LAG-3 expression was related to PD-1 expression on TILs, as well as, PD-L1 expression on tumor cells (TCs) and TILs. Both the univariate and multivariate Cox models indicated that pathological type III (*P* = 0.036), higher LAG-3 on TILs (*P* < 0.001), higher PD-L1 on TCs (*P* = 0.027), and higher PD-1 on TILs (*P* < 0.001) were associated with poorer disease-free survival (DFS). However, lower PD-L1 expression on TILs was related to superior DFS only in the univariate Cox analyses (*P* = 0.002).

**Conclusion:**

Higher LAG-3 and PD-1 on TILs, and higher PD-L1 expression on TCs, and pathological type III were identified as independent risk factors for poorer DFS in NPC patients. Our data demonstrate that LAG-3 is a promising inhibitory receptor that may play an important role in anti-NPC therapy.

**Supplementary Information:**

The online version contains supplementary material available at 10.1186/s12935-021-02162-w.

## Background

Nasopharyngeal carcinoma (NPC) is a common malignancy of the upper or side wall of the nasopharyngeal chamber. Somewhat unexplainably, NPC has distinct disparities in its geographical distribution, with a particularly high occurrence in Guangdong province, China [1]. NPC is closely related to the Epstein-Barr virus (EBV), with the EBV proven as its main etiologic cause [2]. With recent developments in radiotherapy and combined chemoradiotherapy, the survival of NPC patients has been substantially prolonged [3, 4]. Despite advances in treatment, local relapse and distant metastasis continue to represent major causes of cancer progression following anti-NPC therapy [5]. Due to limited advances made regarding chemotherapy regimens, it is critical to explore novel approaches for the treatment of metastatic NPC that alleviate toxicity and promote survival benefits.

Immune checkpoints expressed on tumor cells and immune cells play a crucial role in inhibiting or enhancing anti-tumor immunity. Blocking immune checkpoints has become a promising anti-neoplastic strategy [6, 7]. Recently, immunotherapeutic strategies targeting programmed cell death-1 (PD-1), and programmed cell death ligand-1 (PD-L1) checkpoints have been associated with a remarkable anti-tumor response among various solid tumors [8]. An increasing number of clinical trials involving immunotherapy have shown promising outcomes for NPC patients [6, 9]. PD-L1 has been shown to adjust type 1 T helper (Th1) autoimmune reactions, and is expressed on both tumor cells (TCs) and tumor-infiltrating lymphocytes (TILs) [10]. PD-L1 promotes tumor cell apoptosis by activating PD-1 expressed on T lymphocytes [11]. PD-1 is a suppressive receptor located on activated T lymphocytes that regulates immunological suppression and immune escape [12]. Inhibiting PD-1 or PD-L1 signaling is a potential therapeutic strategy to strengthen the immune response towards tumor cells. Numerous promising predictive biomarkers for immunotherapy have been suggested for various cancer types, including PD-L1 [13, 14], TILs [15], level of microsatellite instability (MSI) [16], and tumor mutational burden (TMB) [17].

Recently, anti-PD-1 and PD-L1 treatments have been associated with potential clinical effects in some NPC patients [18–22]. However, other related studies have found that immuno-monotherapy was ineffective in NPC patients. For instance, a phase I study showed that the overall response of patients with advanced NPC treated with camrelizumab therapy was 34% (95% CI 24–44) and the median progression-free survival (PFS) was only 5.6 months (95% CI 3.3–7.9) [23]. Another study reported that the median PFS of pembrolizumab monotherapy was only 3.7 months, (95% CI 2.1–13.4) and only 2.8 months (95% CI 1.8–7.4) following treatment with nivolumab monotherapy as palliative treatment for NPC [6, 24]. It has also been reported that only 25% of NPC patients will benefit from anti-PD-1/PD-L1 immunotherapy, highlighting the critical need for further research into novel therapeutic regimens [25].

Lymphocyte activating gene 3 (LAG-3, also termed CD223), is a 51-KD transmembrane protein and a member of the immune globulin superfamily [26]. It represents another potential therapeutic target. It is mainly expressed on natural killer cells [26], B cells [27], TILs [28], and dendritic cells [29, 30]. LAG-3 was first identified in the 1990s, its structure is similar to CD4 as both have four extracellular domains [26]. The *LAG-3* gene is located near the *CD4* gene on chromosome 12, and about 20% of the amino acid sequence of LAG-3 and CD4 are identical. Hence, LAG-3 acts as a ligand to bind to the major histocompatibility complex (MHC) class II, and possesses an even higher affinity than CD4 [31–33]. Pre-clinical studies indicate that LAG-3 inhibition activates the effector capabilities of T cells and synergizes with other immune checkpoint inhibitors (i.e., anti-PD-1/PD-L1) [34–38], which provides a strong rationale for simultaneously targeting LAG-3 and PD-1/PD-L1 to enhance anti-tumor T cell immunity. The upregulation of LAG-3 in TILs and in MHC II+ tumors that are resistant to anti-PD-1 antibodies supports this idea [39]. An increasing number of basic and clinical studies have begun to adopt a LAG-3 blockade strategy. As of March 2021, 14 anti-cancer drugs target LAG-3 (data source: https://www.clinicaltrials.gov). Moreover, a number of pharmaceutical companies in China have distributed LAG-3 fusion proteins, antibodies, and bispecific antibodies targeting LAG-3 and other immune checkpoints. These companies have also applied for clinical applications to enhance the efficacy of immunotherapy (data source: https://www.cde.org.cn/). A previous study indicated that LAG-3 expression is closely related to a worse survival of patients with non-small cell lung cancer (NSCLC) [25]. Conversely, several studies indicate that LAG-3 expression is related to a better survival for gastric cancer [40] and breast cancer patients [41]. However, it remains unclear whether LAG-3 has a significant influence on the prognosis of NPC patients.

It has been reported that LAG-3 can induce T cell dysfunction in the tumor microenvironment (TME) [42–44]. Previous studies have shown that the TME interaction with LAG-3 on TILs can modulate an anti-cancer immunoreaction [45]. The extent of TILs infiltration in the TME is related to the treatment effects of PD-1/PD-L1 inhibition [18, 46]. A recent study indicated that lower CD3+ TIL infiltration was related to a poorer DFS for NPC patients [47]. It is well established that the immune system can eradicate infected or transformed cells, which is largely mediated through the activities of natural killer (NK) and cytotoxic T lymphocyte (CTL) cells [48]. The main mechanism of cellular apoptosis induced by NK cells, and CTLs is through the release of the granzyme B protein (GZMB) [49]. GZMB is localized inside endosomes as a zymogen and is subsequently activated by cathepsins to produce the fully active form of GZMB [50]. A meta-analysis found that GZMB+ lymphocytes were significantly associated with a better overall survival (OS) for patients with hepatocellular carcinoma (HCC) [51] and colorectal cancer [52]. However, the clinical relevance of PD-1, TILs, GZMB, PD-L1, and LAG-3 in patients with NPC remains unclear.

Here, we explore the association between LAG-3 expression and clinical characteristics at the cellular level and in tumor samples from NPC patients. We also evaluate the relationship between PD-1, GZMB, CD3+ TILs, PD-L1 expression and the prognosis of NPC patients.

## Methods

### Patients

This retrospective study included 182 patients who were pathologically diagnosed with NPC between January 1, 2006 and December 30, 2018 at Sun Yat-sen University Cancer Center (SYSUCC), China. Malignancy stages were determined according to the tumor node metastasis (TNM) staging method (eighth version) of the American Joint Committee on Cancer (AJCC). Qualified patients were 18–80 years of age with pathologically diagnosed NPC, no second primary cancer, and no distant metastases. We collected patient information regarding age, gender, smoking history, EBV status, family history, TNM stage, pathologic types, and treatment.

### Cell lines

A tissue microarray (TMA) (2 mm) involving 15 NPC cell lines was generated in the laboratory, and all specimens were evaluated in triplicate. Cells were collected, settled for one night, compounded with 0.9% Sepharose, and allowed to curdle at room temperature for at least 5 min. Each solidified agar pellet was lightly positioned in a cassette and placed in 70% alcohol. The solidified agar pellets were disposed of and implanted in paraffin blocks. The cores were then taken from each encased block to produce the TMA, from which 4-mm sections were sliced.

### Immunohistochemical analysis for LAG-3, CD3, GZMB, PD-L1, and PD-1 expression

Pathologically identified, formalin fixed, paraffin-embedded NPC specimens from patients who were biopsied at SYSUCC were retrospectively tested. Archived hematoxylin–eosin staining sections were assessed by two independent pathologists. Immunohistochemical (IHC) staining for LAG-3, CD3, GZMB, PD-L1, and PD-1 expression was conducted using sections obtained from the formalin-fixed diagnostic specimens. Briefly, 4-µm sections were deparaffinized in xylene, rehydrated, and then treated with a citrate antigen restore buffer (pH 9.0) to expose the antigen in the sections. After processing following the conventional steps, the slides were incubated overnight at 4 °C with primary antibodies against LAG-3 (1:200, ab101500, Abcam, Cambridge, MA), CD3 (1:200, ab16669, Abcam, Cambridge, MA), GZMB (1:100, ab255598, Abcam, Cambridge, MA), PD-L1 (M365329, Dako, Carpenteria, CA), and PD-1 (1:50, 315M, Cell Marque, Rocklin, CA). After washing them three times with a phosphate-buffered saline (PBS), 5  min per wash, the sections were sequentially incubated with a Horseradish peroxidase (HRP)-conjugated goat anti-human secondary antibody (PV6000, ZSGB-BIO, Beijing, China). An evaluation was performed using 3, 3′-Diaminobenzidine (DAB) substrate kits (ZLI-9017, ZSGB-BIO, Beijing, China). The sections were stained with hematoxylin for 4 min and counterstained with bluing reagent for 4 min. The slides were washed and then dehydrated in 70% to 100% alcohol baths followed by xylene baths before coverslip application.

### Pathological evaluation of LAG-3, CD3+ TILs, GZMB, and PD-1/PD-L1 expression

The evaluation of LAG-3, CD3+ TILs, and GZMB, PD-1, and PD-L1 expression in the smears were performed by two pathologists who were blinded to the results of the whole tumor sections. Five randomly selected high-power sites (400×) in every sample were chosen to estimate the number of positive cells. The expression of LAG-3, PD-1, GZMB, and CD3+ TILs was assessed in the tumor stroma only, and PD-L1 staining was assessed in both the tumor and stromal cells [53].

### Confirmation of the LAG-3, GZMB, CD3, PD-1, and PD-L1 expression cut-off using X-tile

The expression score of PD-L1 on TCs was determined by multiplying the intensity and density. The intensity of PD-L1 expression on TCs was scored as 0 (negative), 1 (weak), 2 (moderate), or 3 (strong). The density of PD-L1 expression on TCs was scored as 0 (negative), 1 (0–1%), 2 (2–10%), 3 (11–50%), or 4 (> 50%) [54]. The density of PD-L1 expression on TILs was scored as 0 (< 5%), 1 (5–25%), 2 (26–50%), 3 (51–75%), or 4 (> 75%) [55]. We used X-tile Software (Yale University, New Haven, CT, USA) to evaluate the most suitable cut-off values for LAG-3, GZMB, CD3, PD-1, and PD-L1 and the optimal values for predicting DFS. X-tile Software provides a precise statistical estimation by distributing all cases into two groups based on the "low" or "high" expression of a particular biomarker [56].

### Follow-up

Patients were followed up every 3 months for the first 3  years, and every 6 months over the next 2  years, and finally once each year thereafter. A semiannual follow-up was conducted until the end of the study or the death of the patient, whichever occurred first. The last follow-up time for all living patients was October 2020.

### Statistical analysis

The statistical analyses were conducted using SPSS version 22 (IBM, Armonk, NY, USA) and GraphPad Prism 8 (GraphPad Software, La Jolla, CA, USA). Optimal cut-off values for the biomarkers were obtained using X-tile Software. Chi-square tests and Fisher’s exact tests were used to estimate the relationship between LAG-3 and clinical characteristics, CD3, GZMB, PD-L1, and PD-1. The odds ratios (ORs) for LAG-3 expression were estimated for the variables: age, EBV status, family history, gender, smoking status, pathological pattern, TNM staging, CD3, GZMB, PD-L1, and PD-1. Survival was evaluated using the Kaplan–Meier approach. A Cox regression analysis was used to explore the correlation between the clinicopathological variables, the above biomarkers, and DFS. A *P*-value less than 0.15 in the univariate analysis was used to screen the values eligible for the multivariate analysis with the Cox proportional hazard model, together with 95% confidence intervals (CI). A *P* < 0.05 for all statistics was considered significant.

## Results

### LAG-3 expression in NPC cell lines

**Fig. 1:**
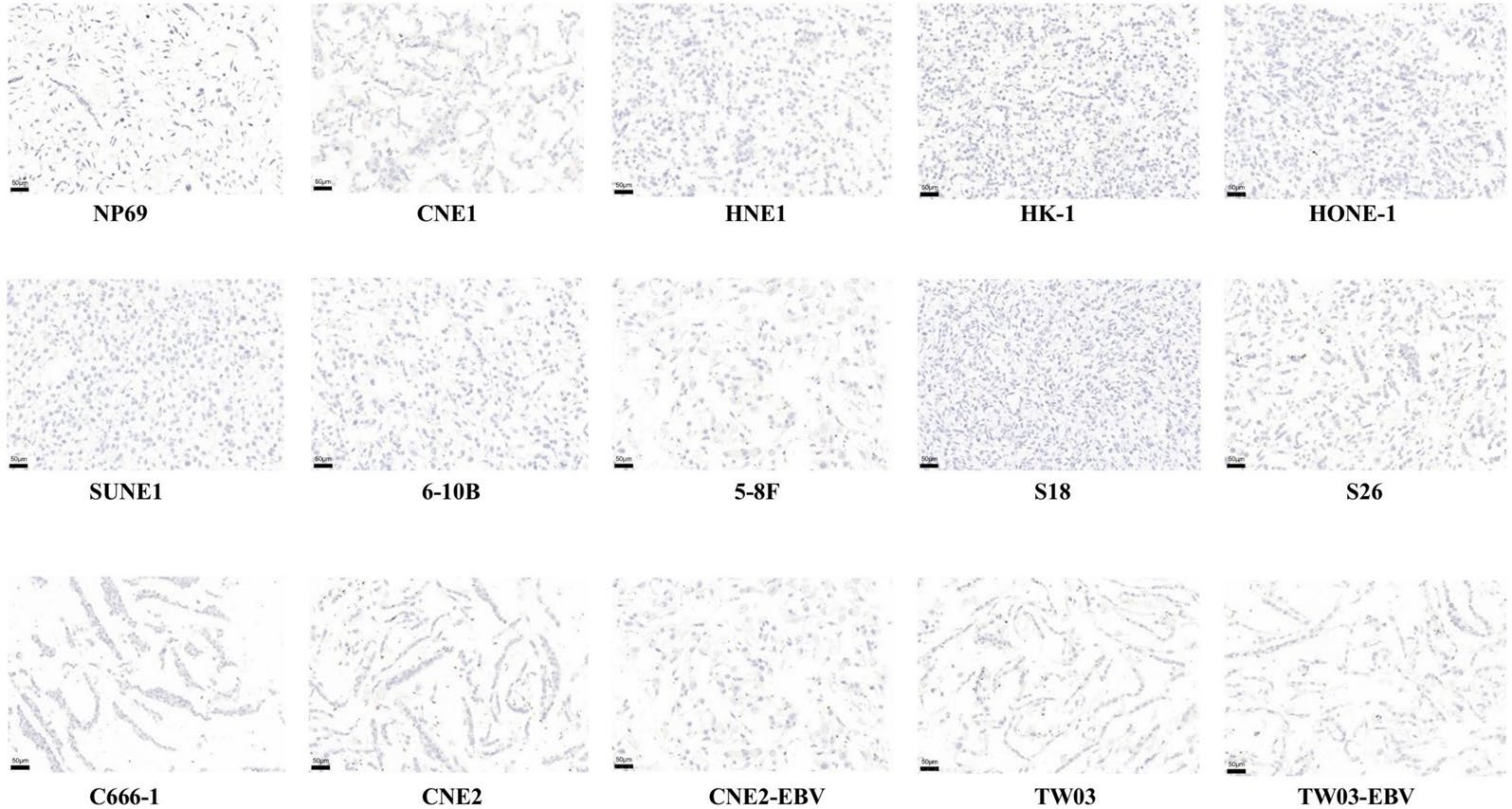
Negative lymphocyte activating 3 (LAG-3) expression in all 15 nasopharyngeal carcinoma (NPC) cell lines (NP69, CNE1, HNE1, HK-1, HONE-1, SUNE1, 6-10B, 5-8F, S18, S26, C666-1, CNE2, CNE2-EBV, TW03, and TW03-EBV) (×20). Scale bars: 50 μm

The hematoxylin–eosin (HE) staining results for 15 NPC cell lines (NP69, CNE1, HNE1, HK-1, HONE-1, SUNE1, 6-10B, 5-8F, S18, S26, C666-1, CNE2, CNE2-EBV, TW03, and TW03-EBV) are summarized in Additional file 1: Figure S1. The expression of LAG-3 was not examined in all of the 15 NPC cell lines (Fig. 1).

### Patient clinical characteristics

**Fig. 2:**
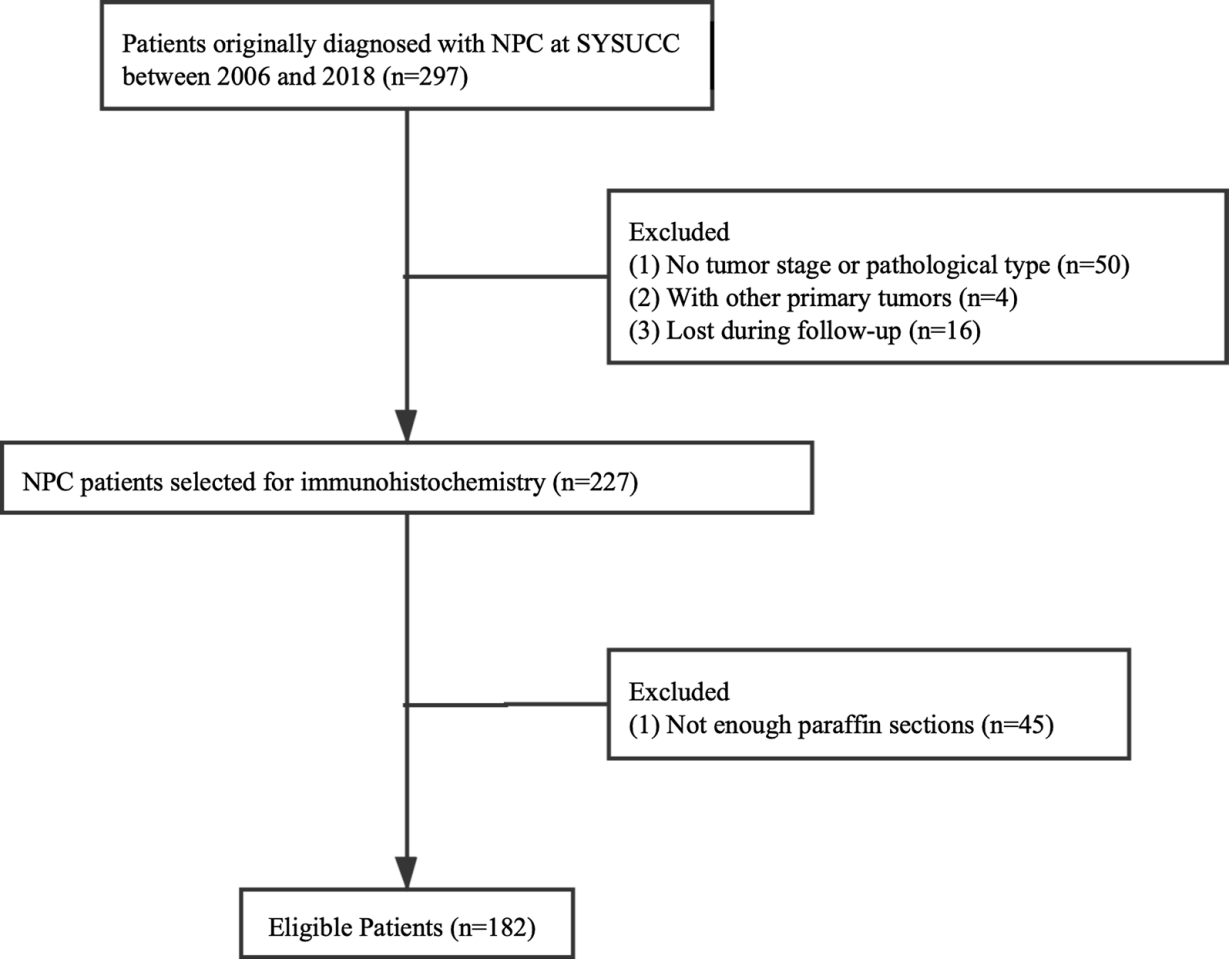
Patient identification and randomization. 297 patients originally diagnosed with NPC were screened according to the inclusion criteria and exclusion criteria, 115 patients were excluded, of which 50 patients had no tumor staging or pathological type, four patients had second primary tumors, and 16 patients were lost to follow-up, and 45 patients had insufficient paraffin sections. Finally, 182 patients diagnosed with NPC were reviewed

In this study, 297 patients originally diagnosed with NPC at SYSUCC were screened for eligibility, 115 patients did not meet the inclusion criteria, of which 50 patients had no tumor staging or pathological type, four patients had other primary tumors, and 16 patients were lost to follow-up, and 45 patients had insufficient paraffin sections. Therefore, 182 NPC patients were evaluated using a series of screening steps (Fig. 2). The median follow-up time was 23.2 months in this patient population, and 50 (27.5%) patients were females and 132 (72.5%) were males. A total of 115 (63.2%) patients were EBV positive, 67 (36.8%) were EBV-negative. The patients’ age ranged from 24 to 76 years old, with a median age of 49 years old. A total of 26 (14.3%) patients had a family history of NPC. Most patients were pathological type III (69.8%), only 14 (7.7%) and 41 (22.5%) patients were pathological type I and II, respectively. There were 31 (17%) smokers and 151 (83%) nonsmokers. The cancer stages were as follows: stage I, nine (4.9%); stage II, 35 (19.2%); stage III, 96 (52.7%); and stage IV, 42 (23.2%). Forty (22%) patients were treated with induction chemotherapy, 11 (6%) with radiotherapy, and 130 (71.4%) with radiochemotherapy (Table 1).

**Table 1:** Characteristics of all patients (n = 182) (100%)

Characteristics	Cases (n = 182)	Percentage (%)
Age (years)		
Median (range)	49 (24–76)	
< 60	156	85.7
≥ 60	26	14.3
Gender		
Male	132	72.5
Female	50	27.5
Smoking status		
Non-smoker	151	83
Smoker	31	17
EBV status		
Positive	115	63.2
Negative	67	36.8
Family history		
Yes	26	14.3
No	156	85.7
Pathological type		
I	14	7.7
II	41	22.5
III	127	69.8
T-stage		
T1	24	13.2
T2	53	29.1
T3	72	39.6
T4	33	18.1
N-stage		
N0	34	18.7
N1	60	33
N2	76	41.8
N3	12	6.5
M-stage		
M0	178	97.8
M1	4	2.2
Disease stage		
I	9	4.9
II	35	19.2
III	96	52.7
IV	42	23.2
DFS (months)		
Median (range)	23.2 (4.6–156.3)	
Treatment		
Induction chemotherapy	40	22
Radiotherapy	11	6
Radiochemotherapy	130	71.4
Chemotherapy	1	0.6

DFS: disease-free survival

### Evaluation of all biomarkers using X-tile

**Fig. 3:**
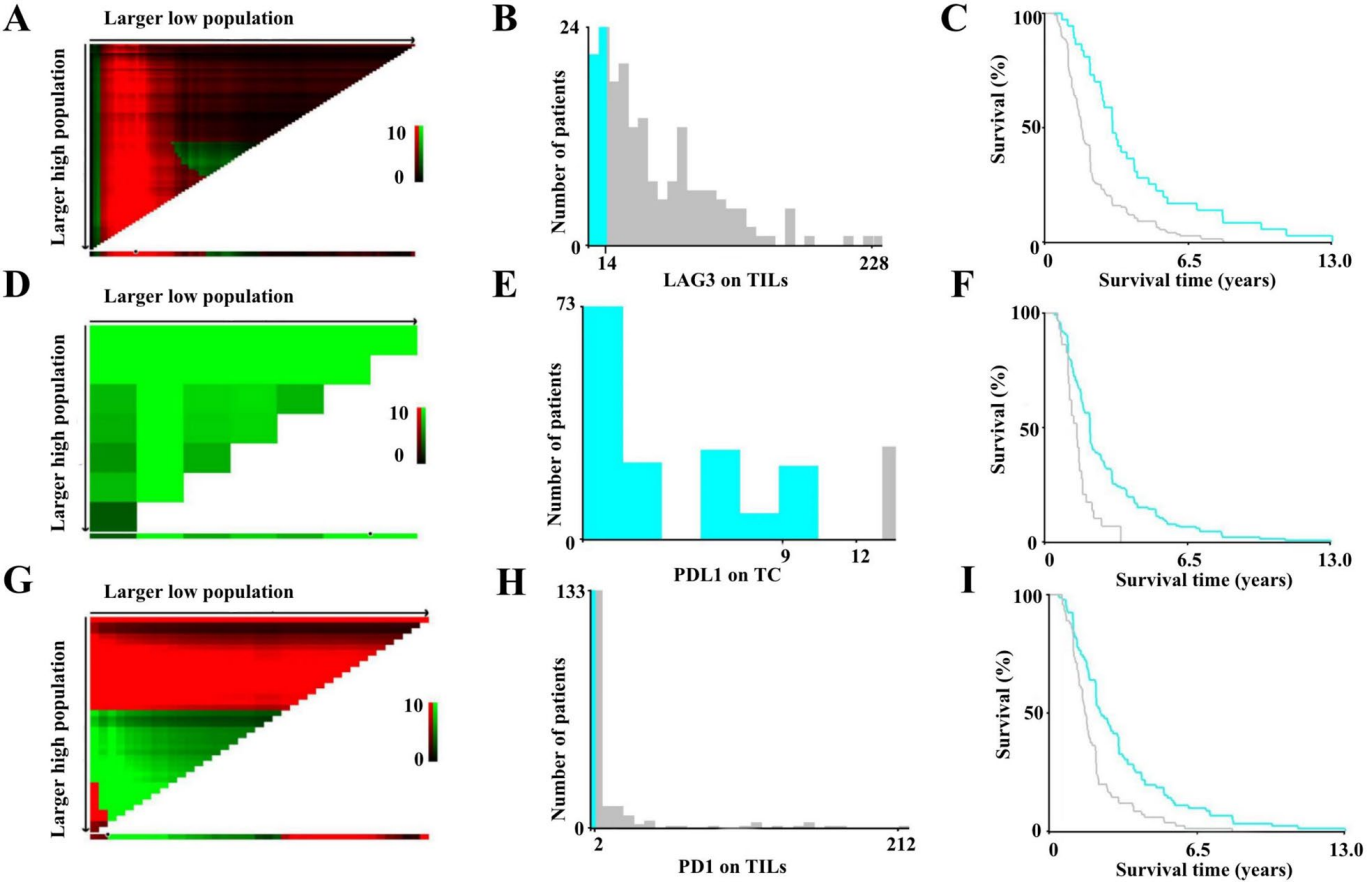
Determination of cut-off values for LAG-3 expression on TILs, PD-L1 expression on TC, PD-1 expression on TILs and survival analyses. X-tile analysis of DFS was performed using patient data to evaluate the optimal cut-off values for LAG-3, PD-L1 and PD-1 expression. The optimal cut-off values are highlighted by the black circles in the left panels (**A**, **D**, **G**) and are shown in the histograms of the entire cohort (middle panels (**B**, **E**, **H**)), and Kaplan–Meier plots are displayed in the right panels (**C**, **F**, **I**). The optimal cut-off value for LAG-3 expression on TILs was 14, patients with LAG-3 expression on more than 14 cells were associated with poorer disease-free survival (DFS) than patients with LAG-3 expression on fewer than 14 cells (**A**–**C**). The optimal cut-off value for PD-L1 expression on TC was nine, patients with PD-L1 on TCs with a score lower than nine had better survival than those with PD-L1 on TCs with a score higher than nine (**D**, **E**). The optimal cut-off value for PD-1 expression on TILs was two cells, patients with PD-1 expression on fewer than two cells was related to superior DFS than those with PD-1 expression on more than two cells (**G**, **H**)

The optimal cut-off values for LAG-3 on TILs, PD-L1 on TCs, and PD-1 expression on TILs obtained using X-tile were 14 cells **(**Fig. 3A, B), 9 (Fig. 3D, E), and 2 cells (Fig. 3G, H), respectively. Higher expression of LAG-3 and PD-1 on TILs as well as PD-L1 expression on TCs were associated with shorter a DFS compared with those with a lower expression than their respective cut-off values (Fig. 3C, F, I). However, the appropriate cut-off values for GZMB, CD3, and PD-L1 expression on TILs obtained by X-tile were unassociated with obvious statistical differences, and consisted of 112 cells, 215 cells, and 1%, respectively (Table 4).

### Expression of LAG-3, CD3, GZMB and PD-1, and PD-L1 in NPC and their correlation with clinical characteristics

**Fig. 4:**
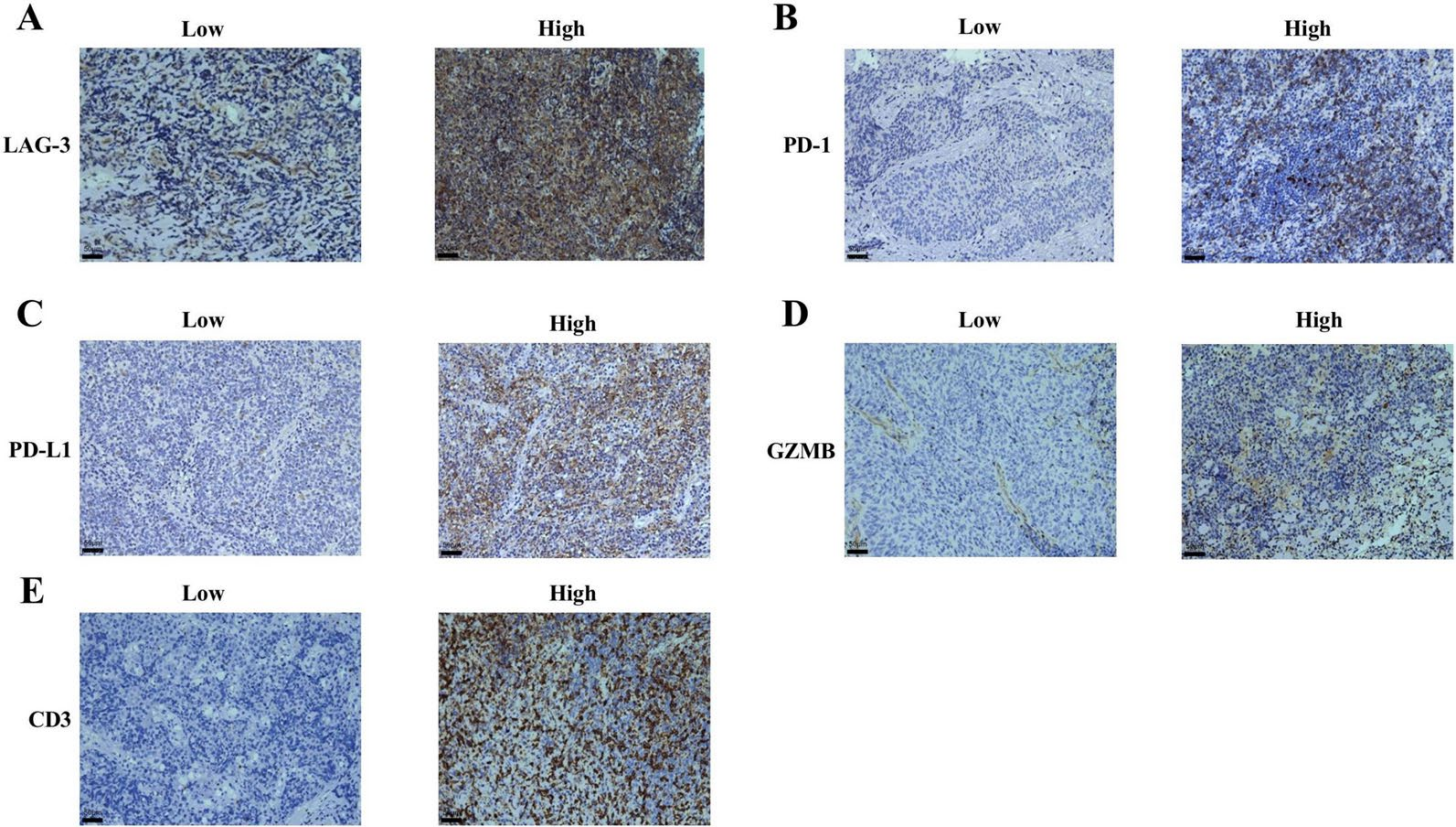
Positive immunohistochemical staining for LAG-3, PD-1, PD-L1, GZMB, and CD3 expression in NPC patients (×20). IHC staining demonstrated low and high expression of LAG-3 (**A**), PD-1 (**B**), PD-L1 (**C**), CD3 (**E**) in the cell membranes and GZMB in the cytoplasm (**D**). Brown staining represents positive cell expression, the number of positive stained cells was manually counted in the formalin fixed paraffin-embedded tissue area (i.e., each 4-µm section). Five randomly selected high-power sites (×400) in every sample were chosen to estimate the number of positive cells. Scale bars: 50 μm

IHC staining demonstrated low and high expression of LAG-3 (Fig. 4A), PD-1 (Fig. 4B), PD-L1 (Fig. 4C), CD3 (Fig. 4E) in the cell membranes and GZMB in the cytoplasm (Fig. 4D). LAG-3 expression was high in 147 (80.8%) patients. There were 90 (49.5%) patients who exhibited high PD-1 expression and 154 (84.6%) patients with high PD-L1 expression on TILs. Thirty-nine (21.4%) patients had high PD-L1 expression on TCs. Further evaluation of TILs found that 125 (68.7%) patients had high CD3 expression, and 44 (24.2%) patients had high GZMB expression (Table 2). Male patients (χ^2^ = 5.147, OR = 2.400, 95% CI 1.112–5.181, *P* = 0.023) with a positive-EBV status (χ^2^ = 15.560, OR = 4.487, 95% CI 2.052–9.809, *P* < 0.001) presented higher LAG-3 expression (Table 3).

**Table 2:** Expression of LAG-3, PD-1, and PD-L1, CD3, GZMB in NSCLC patients

Characteristics	Cases (n = 182)	Percentage (%)
LAG-3 expression on TILs		
High	147	80.8
Low	35	19.2
PD-1 expression on TILs		
High	90	49.5
Low	92	50.5
PD-L1 expression on TC		
High	39	21.4
Low	143	78.6
PD-L1 expression on TILs		
High	154	84.6
Low	28	15.4
CD3+ TIL		
High	125	68.7
Low	57	31.3
GZMB		
High	44	24.2
Low	138	75.8

LAG-3: lymphocyte activating 3; PD-L1: programmed death ligand 1; PD-1: programmed death 1; TIL: tumor-infiltrating lymphocyte; TC: tumor cell; GZMB: granzyme B; NSCLC: non-small cell lung cancer

**Table 3:** Relationships between LAG-3 and clinical data

Characteristic	LAG-3 expression on TILs		
	≤ 14	> 14	*p* value
Age, n (%)			
< 60	27 (14.8)	128 (70.3)	0.137
≥ 60	8 (4.5)	19 (10.4)	
Gender, n (%)			
Female	15 (8.2)	35 (19.2)	**0.023**
Male	20 (11)	112 (61.6)	
Smoking status, n (%)			
Non-smoker	27 (14.8)	124 (68.1)	0.308
Smoker	8 (4.4)	23 (12.7)	
Disease stage, n (%)			
Stage I–II	7 (3.8)	37 (20.3)	0.521
Stage III–IV	28 (15.5)	110 (60.4)	
Pathological type, n (%)			
I–II	8 (4.4)	47 (25.8)	0.291
III	27 (14.9)	100 (54.9)	
EBV status, n (%)			
Negative	23 (7.1)	44 (29.7)	** < 0.001**
Positive	12 (12.1)	103 (51.1)	
Family history, n (%)			
Yes	8 (4.4)	18 (9.9)	0.107
No	27 (14.8)	129 (70.9)	
Treatment, n (%)			
Induction chemotherapy	5 (2.7)	35(19.2)	0.213
Radiotherapy	3 (1.6)	8 (4.5)	
Radiochemotherapy	26 (14.3)	104 (57.2)	
Chemotherapy	1(0.5)	0 (0)	

All the *p *values marked in bold are less than 0.05, which is statistically significant

LAG-3: lymphocyte activating 3; TIL: tumor-infiltrating lymphocyte; EBV: Epstein-Barr virus

### Correlation between LAG-3 expression and other immune checkpoints

A close relationship was observed among LAG-3, PD-1, and PD-L1 expression. We also conducted a relativity analysis between LAG-3, GZMB, and CD3 TILs. High LAG-3 expression was significantly related to high PD-1 expression on TILs (χ^2^ = 5.630, OR = 2.535, 95% CI 1.157–5.551, *P* = 0.018), PD-L1 on TCs (χ^2^ = 8.877, OR = 0.307, 95% CI 0.138–0.685, *P* = 0.003), and PD-L1 on TILs (χ^2^ = 8.569, OR = 3.505, 95% CI 1.462–8.404, *P* = 0.003). However, the same correlation was not observed between LAG-3 and CD3+ TILs (*P* = 0.101), or GZMB (*P* = 0.128) expression (Table 4).

**Table 4:** Relationships between different checkpoints

Characteristics	LAG-3 expression on TILs		
	High (> 14)	Low (≤ 14)	*p* value
PD-1 expression on TILs, n (%)			
High (> 2)	79 (43.4)	11 (6.0)	**0.018**
Low (≤ 2)	68 (37.4)	24 (13.2)	**(1.157–5.5)**
PD-L1 expression on TC, n (%)			
High (> 9)	25 (13.7)	14 (7.7)	**0.003**
Low (≤ 9)	122 (67)	21 (11.6)	
PD-L1 expression on TILs, n (%)			
High (> 1%)	130 (71.4)	24 (13.2)	**0.003**
Low (≤ 1%)	17 (8.9)	11 (6.5)	
CD3+ TIL, n (%)			
High (> 215)	105 (57.7)	20 (11)	0.101
Low (≤ 215)	42 (23.1)	15 (8.2)	
GZMB, n (%)			
High (> 112)	39 (21.4)	5 (3.3)	0.128
Low (≤ 112)	108 (58.8)	30 (16.5)	

All the *p* values marked in bold are less than 0.05, which is statistically significant

LAG-3: lymphocyte activating 3; CI: confidence interval; PD-L1: programmed death ligand 1; PD-1: programmed death 1; TIL: tumor-infiltrating lymphocyte; TC: tumor cell; GZMB: granzyme B

### Logistic regression model analysis to predict LAG-3 expression

The calculated ORs for LAG-3 expression were 2.535 (95% CI 1.157–5.551) and 0.513 (95% CI 0.225–1.170) when low PD-1 expression was compared with high PD-1 expression on TILs and low PD-L1 expression was compared with high PD-L1 expression on TILs in the logistic regression model univariate analysis. The calculated ORs for LAG-3 expression were 0.271 (95% CI 0.105–0.695) and 3.439 (95% CI 1.280–9.237) when low PD-L1 expression on TCs was compared with high expression, and low PD-L1 expression on TILs was compared with high expression in the logistic regression model multivariate analysis (Table 5).

**Table 5:** Univariate and multivariate analysis for prediction of LAG-3 expression in all patients

Variables	Univariate			Multivariate		
	OR	95% CI	*p* value	OR	95% CI	*p* value
Age (< 60 y vs. ≥ 60 y)	0.501	0.199–1.263	0.143	0.604	0.214–1.703	0.131
Sex (female vs. male)	1.757	0.805–3.833	0.157			
Smoking status (nonsmoker vs. smoker)	0.990	0.372–2.636	0.985			
Pathological type (I–II vs. III)	0.630	0.266–1.492	0.294			
EBV status (negative vs. positive)	0.632	0.282–1.413	0.263			
Family history (yes vs. no)	2.123	0.837–5.384	0.113	2.238	0.786–6.373	0.131
Disease stage (I–II vs. III–IV)	0.743	0.300–1.843	0.522			
PD-1 expression on TILs (Low vs. High)	2.535	1.157–5.551	**0.020**	2.282	0.970–5.367	0.059
PD-L1 expression on TC (Low vs. High)	2.400	0.684–8.418	0.113	0.271	0.105–0.695	**0.007**
PD-L1 expression on TILs (Low vs. High)	0.513	0.225–1.170	**0.005**	3.439	1.280–9.237	**0.014**
CD3**+ **TIL (Low vs. High)	1.875	0.878–4.006	0.105	1.317	0.546–3.175	0.540
GZMB (Low vs. High)	2.167	0.785–5.979	0.135	1.476	0.482–4.520	0.495

All the *p *values marked in bold are less than 0.05, which is statistically significant

LAG-3: lymphocyte activating 3; CI: confidence interval; PD-L1: programmed death ligand 1; PD-1: programmed death 1; TIL: tumor-infiltrating lymphocyte; TC: tumor cells; GZMB: granzyme B; EBV: Epstein-Barr virus; CI: confidence interval; OR: odds ratio

### The impact of LAG-3 and other checkpoints on DFS

**Fig. 5:**
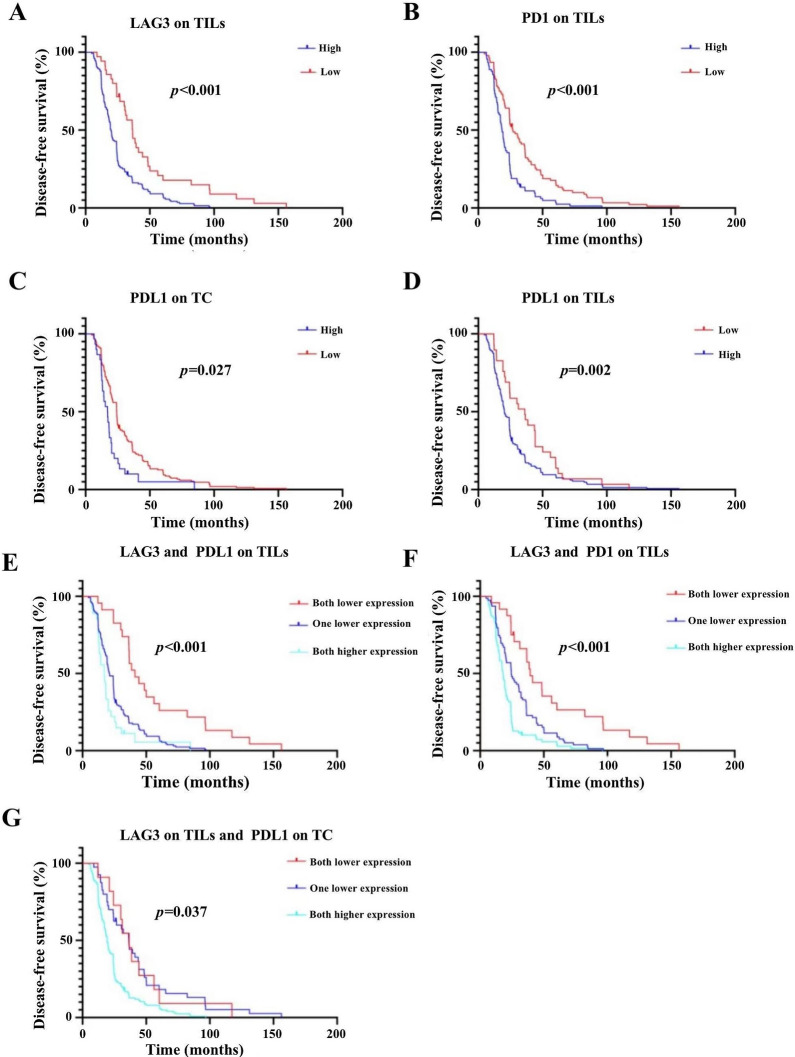
The survival curves of LAG-3, PD-1, and PD-L1 on TILs, PDL1 on TCs layered by the cut-off values estimated by X-tile.** A** Disease-free survival (DFS) differed significantly between patients with LAG-3 expression on fewer than 14 cells and those with LAG-3 expression on more than 14 cells (*P* < 0.001). **B** DFS differed significantly between patients with PD-1 expression on fewer than two cells and those with PD-1 expression on more than two cells (*P* < 0.001). **C** DFS differed significantly between patients with PD-L1 on TCs with a score lower than nine and those with PD-L1 on TCs with a score higher than nine (*P* = 0.027). **D** DFS differed significantly between patients with PD-L1 expression on fewer than 1% of TILs **a**nd those with PD-L1 expression on more than 1% of TILs (*P* = 0.002). **E **DFS differed significantly between patients with lower LAG-3 and PD-L1 expression on TILs and those with higher LAG-3 and PD-L1 expression on TILs (*P* < 0.001). **F **DFS differed significantly between patients with lower LAG-3 and PD-1 expression and those with higher LAG-3 and PD-1 expression (*P* < 0.001). **G** DFS differed significantly between patients with lower LAG-3 and PD-L1 expression on TCs and those with higher LAG-3 and PD-L1 expression on TCs (*P* = 0.037)

We found that lower LAG-3, PD-1, and PD-L1 expression was associated with a more favorable survival prognosis. The Kaplan–Meier analysis estimated that higher LAG-3 expression on TILs (19.7 months [95% CI 18.1–24] versus 36.4 months [95% CI 26.7–44.3],* p* < 0.001) (Fig. 5A), PD-1 expression on TILs (18.45 months [95% CI 16.65–20.44] versus 26.55 months [95% CI 24.3–34.6], *p* < 0.001) (Fig. 5B), and PD-L1 expression on TCs (17.15 months [95% CI 13.15–19.4] versus 24.3 months [95% CI 20.7–24.9], *p* = 0.027) (Fig. 5C), as well as, PD-L1 expression on TILs (20.3 months [95% CI 18.3–24.2] versus 36.2 months [95% CI 24.3–44.3], *p* = 0.002) (Fig. 5D) were associated with a distinctly shorter DFS compared with those with lower expression than their respective cut-off values. We also observed that patients with lower LAG-3 and lower PD-L1 on TILs had a longer DFS than patients who had higher PD-L1 on TILs or LAG-3 or both higher PD-L1 on TILs and LAG-3 (41.2 months [95% CI 36.4–60.3] versus 21.1 months [95% CI 19.3–24.3] versus 17.2 months [95% CI 13.1–20.3], *p* < 0.001) (Fig. 5E). Patients with both lower LAG-3 and lower PD-1 on TILs had a longer DFS than patients who had either higher PD-1 or LAG-3 or higher PD-1 and LAG-3 (38.9 months [95% CI 26.7–56.2] versus 24.5 months [95% CI 20.1–30.6] versus 18.1 months [95% CI 15.2–19.7], *p* < 0.001) (Fig. 5F). Patients with both lower LAG-3 and lower PD-L1 on TCs had a longer DFS than patients who had either a higher PD-L1 on TCs or LAG-3 or higher PD-L1 on TCs and LAG-3 (36.6 months [95% CI 24.3–56.2] versus 36.25 months [95% CI 24.35–44] versus 19.3 months [95% CI 17.2–22.3], *p* < 0.001) (Fig. 5G).

### Cox regression analysis for DFS

All univariate and multivariate analyses of prognostic factors are summarized in Table 6. The univariate analysis results indicated that the relevant risk factors for survival were age (≥ 60 vs. < 60 y, HR, 95% CI 1.00 (ref.) vs 1.588 (1.024–2.462), *P* = 0.039), pathological type (I–II vs. III, HR, 95% CI 1.00 (ref.) vs 0.696 (0.495–0.977), *P* = 0.036), LAG-3 expression on TILs (Low vs. High, HR, 95% CI 1.00 (ref.) vs 0.441 (0.296–0.657), *P* < 0.001), PD-1 expression on TILs (Low vs. High, HR, 95% CI 1.00 (ref.) vs 0.494 (0.363–0.671), *P* < 0.001), PD-L1 expression on TCs (Low vs. High, HR, 95% CI 1.00 (ref.) vs 0.659 (0.455–0.953), *P* = 0.027), and PD-L1 expression on TILs (Low vs. High, HR, 95% CI 1.00 (ref.) vs 0.678 (0.452–1.017), *P* = 0.002).

**Table 6:** COX regression analysis of DFS

Variables	Univariate			Multivariate		
	HR	95% CI	*p* value	HR	95% CI	*p* value
Age (≥ 60 vs. < 60 y)	1.588	1.024–2.462	**0.039**	1.222	0.779–1.916	0.382
Sex (Female vs. Male)	1.006	0.717–1.412	0.972			
Smoking status (Nonsmoker vs. Smoker)	1.049	0.708–1.554	0.813			
Pathological type (I–II vs. III)	0.696	0.495–0.977	**0.036**	0.693	0.484–0.992	**0.045**
EBV status (Negative vs. Positive)	0.838	0.614–1.144	0.267			
Family history (Yes vs. No)	0.942	0.619–1.433	0.780			
Disease stage (III–IV vs. I–II)	1.051	0.747–1.479	0.773			
LAG-3 expression on TILs (Low vs. High)	0.441	0.296–0.657	** < 0.001**	0.434	0.284–0.663	** < 0.001**
PD-1 expression on TILs (Low vs. High)	0.494	0.363–0.671	** < 0.001**	0.558	0.400–0.778	**0.001**
PD-L1 expression on TC (Low vs. High)	0.659	0.455–0.953	**0.027**	0.636	0.432–0.937	**0.022**
PD-L1 expression on TILs (Low vs. High)	0.678	0.452–1.017	**0.002**	1.050	0.667–1.654	0.832
CD3+ TIL (Low vs. High)	1.179	0.731–1.900	0.499			
GZMB (Low vs. High)	0.661	0.467–0.936	0.063	1.119	0.757–1.653	0.574

All the *p* values marked in bold are less than 0.05, which is statistically significant

LAG-3: lymphocyte activating 3; PD-L1: programmed death ligand 1; PD-1: programmed death 1; GZMB: granzyme B; TIL: tumor-infiltrating lymphocyte; TC: tumor cells; DFS: disease-free survival; HR: hazard ratio; CI: confidence interval

The multivariate Cox analysis revealed that the independent risk factors for survival were pathological type (I–II vs. III, HR, 95% CI 1.00 (ref.) vs 0.693 (0.484–0.992), *P* = 0.045), LAG-3 expression on TILs (Low vs. High, HR, 95% CI 1.00 (ref.) vs 0.434 (0.284–0.663), *P* < 0.001), PD-1 expression on TILs (Low vs. High, HR, 95% CI 1.00 (ref.) vs 0.558 (0.400–0.778), *P* = 0.001), and PD-L1 expression on TCs (Low vs. High, HR, 95% CI 1.00 (ref.) vs 0.636 (0.432–0.937), *P* = 0.022).

## Discussion

LAG-3 represents a promising immune checkpoint inhibitor and has been investigated as a target for the treatment of solid tumors in many studies [57]. One study found that LAG-3 suppresses antitumor immunity in Hodgkin’s lymphoma [58]. Anti-LAG-3 treatment was also found to restrict breast carcinoma growth in an animal model [35]. Moreover, simultaneously inhibiting LAG-3 and PD-1 signaling can strengthen the T lymphocyte response in ovarian carcinoma [59]. An increasing number of basic research and clinical studies have begun to adopt a LAG-3 blockade strategy in the treatment of solid tumors [60, 61]. However, data regarding LAG-3 expression in NPC and its correlation with TILs, GZMB, PD-1, and PD-L1 remains unclear in NPC patients.

A limited number of studies have investigated LAG-3 expression in NPC and its relationship with TILs, PD-1, PD-L1, and GZMB. In our study, we first examined LAG-3 expression in NPC cell lines as well as clinical specimens and found that LAG-3 was negatively expressed on NPC cell lines regardless of EBV status, but that it was highly expressed on TILs in NPC cancer specimens. Male patients and those who were EBV-positive displayed higher LAG-3 expression. We also discovered that LAG-3 was closely related to PD-1 and PD-L1 expression. A survival analysis demonstrated that NPC patients with lower LAG-3, PD-1, and PD-L1 expression had a longer DFS. Importantly, higher LAG-3, PD-1, and PD-L1 expression on TCs, and pathological type III were confirmed to be independent prognostic factors for poorer DFS in NPC patients.

In regions where NPC is prevalent, it is primarily related to EBV infection status [62]. The World Health Organization (WHO) has identified histological categories of NPC, among which, keratinizing squamous cell malignancy is defined as type I, differentiated non-keratinizing malignancy is defined as type II, and undifferentiated non-keratinizing malignancy is defined as type III, which is closely linked with EBV infection status [63–65]. Our findings indicate that patients who are EBV positive exhibit higher LAG-3 expression. In addition, higher expression of LAG-3 and pathological type III were identified as independent prognostic risk factors for poorer DFS. The correlation between LAG-3 and EBV status may explain the poor prognosis of NPC patients with pathological type III.

Our findings demonstrate that LAG-3 is positively related to PD-L1 expression on TCs and PD-1 expression on TILs, which is in line with the results of previous studies. One study showed that LAG-3 was closely related to PD-1 and PD-L1 expression in NSCLC [25]. LAG-3 and PD-1 can regulate immune activation and synchronously increase immunity [66]. A study investigating ovarian cancer found that LAG-3 and PD-1 can down-regulate TILs [67]. Moreover, it was shown that LAG-3 and PD-1 can synchronously regulate the behavior and anticancer response of T lymphocytes [68]. An in vivo study found that targeting the PD-1 or LAG-3 signaling pathways could stimulate T lymphocytes, and that the combined inhibition of these pathways had a greater effect than the inhibition of each pathway alone [34]. Previous studies have found that LAG-3 protein expression may act synergistically with PD-1 or PD-L1 monoclonal antibodies [68–70]. Anti-LAG-3 therapy has also been shown to effectively modulate regulatory T lymphocytes, [71] whereas other immune checkpoints (e.g., PD-1, PD-L1, and CTLA-4 (cytotoxic T lymphocyte associated antigen-4)) have not. A total of 14 anti-cancer drugs targeting LAG-3 have been developed as of March 2021 (Table 7) (data source: https://www.clinicaltrials.gov). IMP321 was the first anti-cancer drug targeting LAG-3 to enter clinical trials. Clinical research has identified that a double blockade of immune checkpoint molecules results in enhanced clinical survival in various cancers, including renal cell carcinoma [72], melanoma [73], NSCLC [74], and small cell lung cancer [75]. It is important to note that in the reported camrelizumab antibody therapy NPC study, six out of eight patients who had formerly received ipilimumab (anti-CTLA-4) treatment exhibited a clinical response [23]. This finding indicates that a combination of immunotherapeutic strategies warrants further research.

**Table 7:** Clinical trials targeting LAG-3 expression as of March 2021

Antibody name	Targets	Clinical studies	Phase	Conditions
IMP321	LAG-3 Fusion Protein	NCT00732082	Phase I	Pancreatic neoplasms
		NCT03252938	Phase I	Solid tumors
		NCT00351949	Phase I	Stage IV renal cell Carcinoma
		NCT03625323	Phase II	NSCLC, head and neck squamous cell carcinoma (HNSCC)
		NCT04252768	Phase I	Metastatic breast cancer
		NCT00349934	Phase I	Metastatic breast cancer
		NCT02614833	Phase II	Adenocarcinoma breast
		NCT04811027	Phase II	HNSCC
Relatlimab	LAG-3	NCT04080804	Phase II	HNSCC
		NCT01968109	Phase I/IIa	Neoplasms
		NCT02061761	Phase I/IIa	Hematologic neoplasms
		NCT03610711	Phase II	Gastroesophageal cancer
		NCT02658981	Phase I	Glioblastoma
		NCT04150965	Phase I/II	Multiple myeloma
		NCT03044613	Phase Ib	Gastric cancer, Esophageal cancer, gastroesophageal cancer
		NCT04611126	Phase I	Metastatic ovarian cancer, metastatic fallopian tube cancer, peritoneal cancer
		NCT03623854	Phase II	Chordoma
		NCT03459222	Phase I/II	Advanced cancer
		NCT02966548	Phase I	Cancer
		NCT03662659	Phase II	Gastric cancer, cancer of the stomach, esophagogastric Junction
		NCT03743766	Phase II	Melanoma
		NCT04326257	Phase II	Squamous cell carcinoma of the head and neck
		NCT04658147	Phase I	Hepatocellular carcinoma
		NCT03607890	Phase II	Cancer
		NCT04567615	Phase II	Hepatocellular carcinoma
		NCT02519322	Phase II	Melanoma
		NCT02060188	Phase II	Microsatellite unstable colorectal cancer
		NCT03493932	Phase I	Glioblastoma
		NCT02488759	Phase I/II	Advanced cancer
Sym022	LAG-3	NCT03489369	Phase I	Metastatic cancer
		NCT04641871	Phase I	Metastatic cancer
		NCT03311412	Phase I	Metastatic cancer
RO7247669	PD-1 × LAG-3	NCT04140500	Phase I	Solid tumors
		NCT04785820	Phase II	Advanced or metastatic esophageal Squamous cell carcinoma
REGN3767	LAG-3	NCT03005782	Phase I	Malignancies
TSR-033	LAG-3	NCT03250832	Phase I	Neoplasms
EMB-02	PD-1 × LAG-3	NCT04618393	Phase I/II	Advanced solid tumors
MGD013	PD-1 × LAG-3	NCT03219268	Phase I	Advanced solid tumors
		NCT04082364	Phase II/III	Gastric cancer
		NCT04634825	Phase II	Colorectal cancer
		NCT04129320	Phase II/III	Head and neck cancer
FS118	PD-L1 × LAG-3	NCT03440437	Phase I/II	Advanced cancer
INCAGN02385	LAG-3	NCT04370704	Phase I/II	Melanoma
TSR-033	LAG-3	NCT02817633	Phase I	Neoplasms
LAG525	LAG-3	NCT03365791	Phase II	Advanced solid tumors, diffuse large B cell lymphoma
		NCT03742349	Phase I	Triple negative breast cancer
		NCT02460224	Phase I/II	Advanced solid tumors
XmAb-22841	CTLA-4 × LAG-3	NCT03849469	Phase I	Advanced solid tumors
EOC202	LAG-3 fusion protein	NCT03600090	Phase I	Advanced solid tumors

NSCLC: non-small cell lung cancer; HNSCC: head and neck squamous cell carcinoma; LAG-3: lymphocyte activating 3; PD-L1: programmed death ligand 1; PD-1: programmed death 1; CTLA-4: cytotoxic T-lymphocyte antigen 4

The findings of our study indicate that the inhibition of both LAG-3 and PD-1/PD-L1 can enhance the anti-cancer response as part of a synergy. Bispecific antibodies (BsAbs) (e.g., anti-LAG3, PD-1/PD-L1) have been exploited for extensive clinical use. MGD013 is a BsAb therapy that simultaneously targets both LAG-3 and PD-1 to suppress immune checkpoint inhibition, promote T cell activation, and improve anti-cancer immunity. Similarly, F-star exploited a BsAb termed FS118, which simultaneously targets LAG-3 and PD-L1. In addition, numerous pharmaceutical companies in China have created LAG-3 fusion proteins, antibodies, and bispecific antibodies, which are undergoing clinical applications (Table 8) (data source: https://www.cde.org.cn/). Since LAG-3 is closely related to PD-1 and PD-L1, our study provides a novel insight and a theoretical foundation for the future development of LAG-3 and PD-1/PD-L1 bispecific antibodies to enhance the efficacy of immunotherapy for NPC.

**Table 8:** Clinical applications as of March 2021 for bispecific antibodies targeting LAG-3 expression

Antibody name	Targets	Clinical application acceptance number	Date
HLX26	LAG-3	CXSL2100041	2021-02-03
IBI323	PD-L1 × LAG-3	CXSL2000242	2020-08-25
EMB-02	PD-1 × LAG-3	CXSL2100047	2020-05-23
DNV3	LAG-3	CXSL2000121	2020-05-22
KL-A289	LAG-3	CXSL2000108	2019-11-16
MGD013	PD-1 × LAG-3	JXSL1900114	2019-08-23
SHR-1802	LAG-3	CXSL1900090	2019-05-27
LBL-007	LAG-3	JXSL1900040	2019-04-26
IBI110	LAG-3	CXSL1900040	2019-04-26
EOC202	LAG-3	CTR20180185	2018-06-25

LAG-3: lymphocyte activating 3; PD-L1: programmed death ligand 1; PD-1: programmed death 1

We also observed that LAG-3 expression was associated with poor survival, which is in accordance with the results of other studies. One study indicated that high LAG-3 expression was related to worse survival in patients with NSCLC [25]. In chronic lymphocytic leukemia, LAG-3 also serves as a new predictive marker: higher expression of LAG-3 was associated with shorter survival [76]. Yet, in contrast, several studies have found that high LAG-3 was correlated with better survival in patients with gastric carcinoma [40] and breast carcinoma [41]. Thus, LAG-3 represents a potential immune checkpoint target. Despite the numerous ongoing anti-LAG-3 studies, the literature related to LAG-3 and NPC remains insufficient. Consequently, it is necessary to further explore the prognostic value of LAG-3 in NPC. The findings of our present study indicate that LAG-3 may participate in the tumor immune escape of NPC as an interpretation of the observed poor survival in NPC patients. In addition, the association of LAG-3 and GZMB, and CD3+ TIL expression was also analyzed. However, no correlation between LAG-3 and GZMB or CD3+ TIL expression was found. A previous study indicated that lower CD3+ TIL infiltration was related to a worse DFS in patients with NPC [47] and HCC [77], which is inconsistent with our findings. Our finding that CD3 was unrelated to NPC prognosis may be attributed to our comparatively small case size. Further investigation with a larger sample size and an independent cohort of patients is required.

There are some limitations of our study. First, this was a retrospective study, and we only collected information from one institute, and the overall survival data was insufficient for rigorous analysis. Secondly, since previous related reports have not identified the optimal cut-off value for LAG-3, X-tile was used to determine the cut-off values for predicting DFS. Finally, the sample size of this population was small. Further research involving a larger number of NPC patients is required.

## Conclusions

Immune checkpoints play a critical role in immune regulation. Yet, the synergistic effects between multiple immune targets remains unknown. CTLA-4, PD-1, and PD-L1 antibody immunotherapy have demonstrated significant effectiveness for the treatment of some cancers. LAG-3 represents another potential therapeutic target whose synergistic effect requires further investigation. In our present study, we found that LAG-3 was closely associated with PD-1/PD-L1 expression. Positive LAG-3 expression or the expression of both LAG-3 and PD-L1 has been associated with early cancer relapse. Male and EBV-positive patients were associated with higher LAG-3 expression in our study. Lower LAG-3, PD-1, and PD-L1 expression were also associated with a longer DFS. Importantly, high LAG-3, PD-1, and PD-L1 expression on TCs, and Pathological type III were confirmed to be independent risk factors for poorer DFS in NPC patients. Based on the findings of our study and observations supporting its potential synergistic function when administered in conjunction with anti-PD-1/PD-L1, the inhibition LAG-3 is a promising inhibitory receptor and anti-LAG3 will likely play a critical role in anti-neoplastic therapy. Our study provides a theoretical foundation for the exploitation of BsAbs against LAG-3 or PD-1 on TILs, and PD-L1 on TCs. However, to date, there has been minimal research into the synergistic interactions between LAG-3 and other promising immune checkpoint molecules, such as T cell immunoglobulin-3. Future investigations are warranted.

## Supplementary Information

**Additional file 1: Figure S1.** Hematoxylin–eosin (HE) staining of all 15 NPC cell lines (× 20). Scale bars: 50 μm.

## Data Availability

The datasets used and analyzed in this study are available from the corresponding author upon reasonable request. The authenticity of this article has been validated by uploading the key raw data onto the Research Data Deposit public platform (https://www.researchdata.org.cn), with the approval RDD number as RDDA2021002093.

## Abbreviations

NPC: Nasopharyngeal carcinoma
PD-1: Programmed cell death-1
PD-L1: Programmed cell death ligand-1
Th1: T helper
TCs: Tumor cells
TILs: Tumor-infiltrating lymphocytes
MSI: Microsatellite instability
TMB: Tumor mutational burden
PFS: Progression-free survival
LAG-3: Lymphocyte activating gene 3
NSCLC: Non-small cell lung cancer
TME: Tumor microenvironment
NK: Natural killer
CTL: Cytotoxic T lymphocyte
GZMB: Granzyme B
OS: Overall survival
HCC: Hepatocellular carcinoma
MHC: Major histocompatibility complex
HRP: Horseradish peroxidase
DAB, 3: 3′-Diaminobenzidine
PBS: Phosphate-buffered saline
SYSUCC: Sun Yat-sen University Cancer Center
TNM: Tumor node metastasis
AJCC: American Joint Committee on Cancer
EBV: Epstein-Barr virus
TMA: Tissue microarray
IHC: Immunohistochemical
HE: Hematoxylin–eosin
WHO: World Health Organization
CTLA-4: Cytotoxic T lymphocyte associated antigen-4
BsAb: Bispecific antibodies
CI: Confidence interval
OR: Odds ratio
HR: Hazard ratio
DFS: Disease-free survival
