# Correction to: Lymphocyte activating gene 3 protein expression in nasopharyngeal carcinoma is correlated with programmed cell death-1 and programmed cell death ligand-1, tumor-infiltrating lymphocytes

**DOI:** 10.1186/s12935-022-02458-5

**Published:** 2022-01-29

**Authors:** Fan Luo, Jiaxin Cao, Feiteng Lu, Kangmei Zeng, Wenjuan Ma, Yan Huang, Li Zhang, Hongyun Zhao

**Affiliations:** 1 Department of Experimental Research, State Key Laboratory of Oncology in South China, Collaborative Innovation Center for Cancer Medicine, SunYat-Sen University Cancer Center, Guangzhou, China; 2 Department of Medical Oncology, State Key Laboratory of Oncology in South China, Collaborative Innovation Center for Cancer Medicine, Sun Yat-Sen University Cancer Center, 651 Dongfeng Road East, Guangzhou, 510060 Guangdong China; 3 Department of Intensive Care Unit, State Key Laboratory of Oncology in South China, Collaborative Innovation Center for Cancer Medicine, Sun Yat-Sen University Cancer Center, Guangzhou, China; 4 Department of Clinical Research, State Key Laboratory of Oncology in South China, Collaborative Innovation Center for Cancer Medicine, Sun Yat-Sen University Cancer Center, 651 Dongfeng Road East, Guangzhou, 510060 Guangdong China

## Correction to: Cancer Cell Int (2021) 21:458 https://doi.org/10.1186/s12935-021-02162-w

In this article [1], the author would like to correct the two mistakes mentioned below with this erratum.

In the methods section, “LAG-3 (1:200, ab101500, Abcam, Cambridge, MA)” should be changed to “LAG-3 (1:200, ab180187, Abcam, Cambridge, MA)”.

In the Table 2 section, the title “Expression of LAG-3, PD-1, and PD-L1, CD3, GZMB in NSCLC patients” should be changed to “Expression of LAG-3, PD-1, and PD-L1, CD3, GZMB in NPC patients”. Correspondingly, “NSCLC: non-small cell lung cancer” should be changed to “NPC: nasopharyngeal carcinoma” in the caption of the table.
