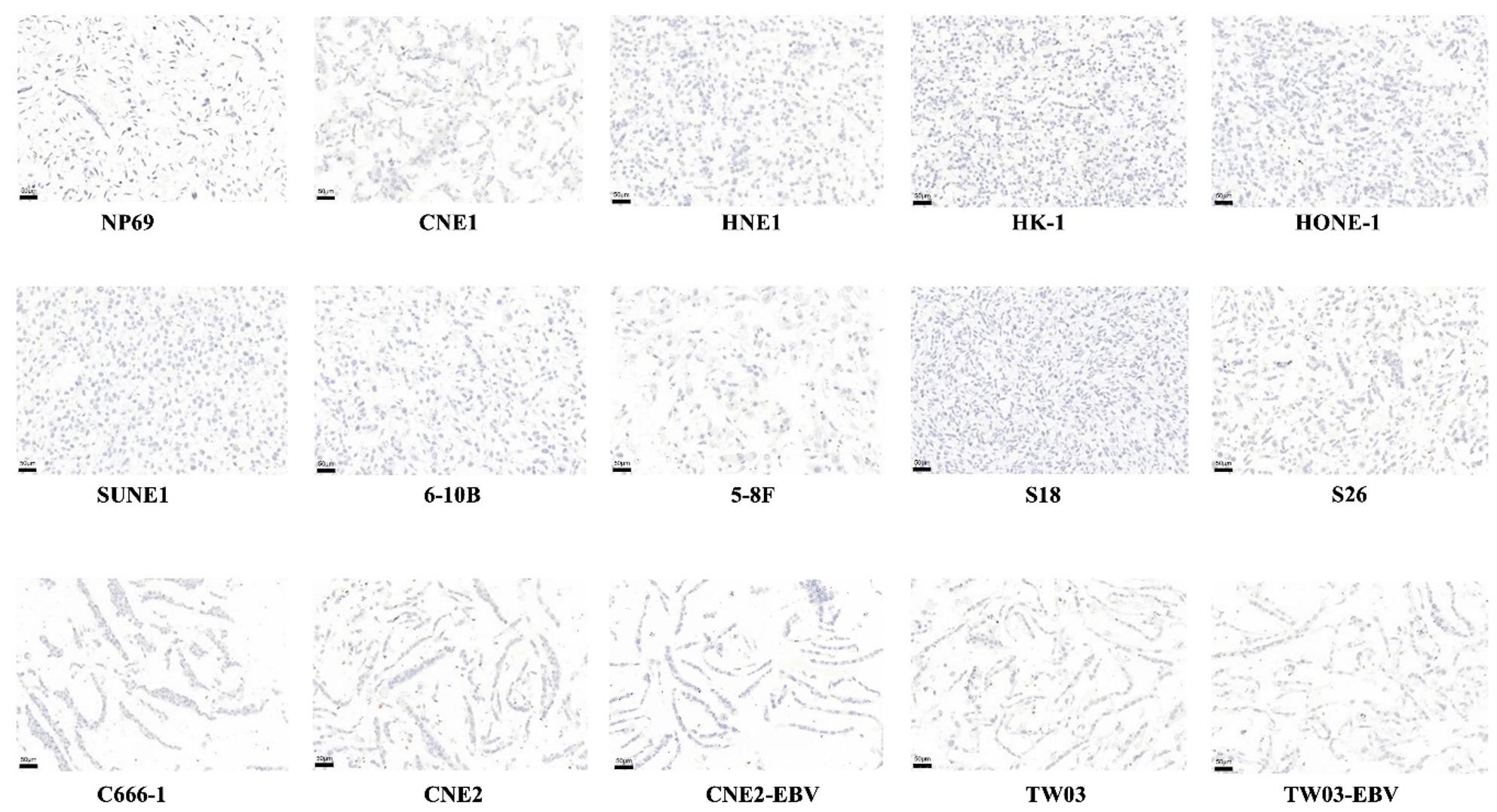# Correction: Lymphocyte activating gene 3 protein expression in nasopharyngeal carcinoma is correlated with programmed cell death-1 and programmed cell death ligand-1, tumor-infiltrating lymphocytes

**DOI:** 10.1186/s12935-026-04350-y

**Published:** 2026-06-17

**Authors:** Fan Luo, Jiaxin Cao, Feiteng Lu, Kangmei Zeng, Wenjuan Ma, Yan Huang, Li Zhang, Hongyun Zhao

**Affiliations:** 1 Department of Experimental Research, State Key Laboratory of Oncology in South China, Collaborative Innovation Center for Cancer Medicine, Sun Yat-Sen University Cancer Center, Guangzhou, China; 2 Department of Medical Oncology, State Key Laboratory of Oncology in South China, Collaborative Innovation Center for Cancer Medicine, Sun Yat-Sen University Cancer Center, 651 Dongfeng Road East, Guangzhou, 510060 Guangdong China; 3 Department of Intensive Care Unit, State Key Laboratory of Oncology in South China, Collaborative Innovation Center for Cancer Medicine, Sun Yat-Sen University Cancer Center, Guangzhou, China; 4 Department of Clinical Research, State Key Laboratory of Oncology in South China, Collaborative Innovation Center for Cancer Medicine, Sun Yat-Sen University Cancer Center, 651 Dongfeng Road East, Guangzhou, 510060 Guangdong China

**Correction: Cancer Cell Int (2021) 21:458**

10.1186/s12935-021-02162-w

In this article [1], The Fig. 1 has 2 similar image in both 58 F and CNE2-EBV and it should have different images. The incorrect and correct versions of Fig. 1 are displayed below. The original article has been corrected.

Incorrect Fig. 1:


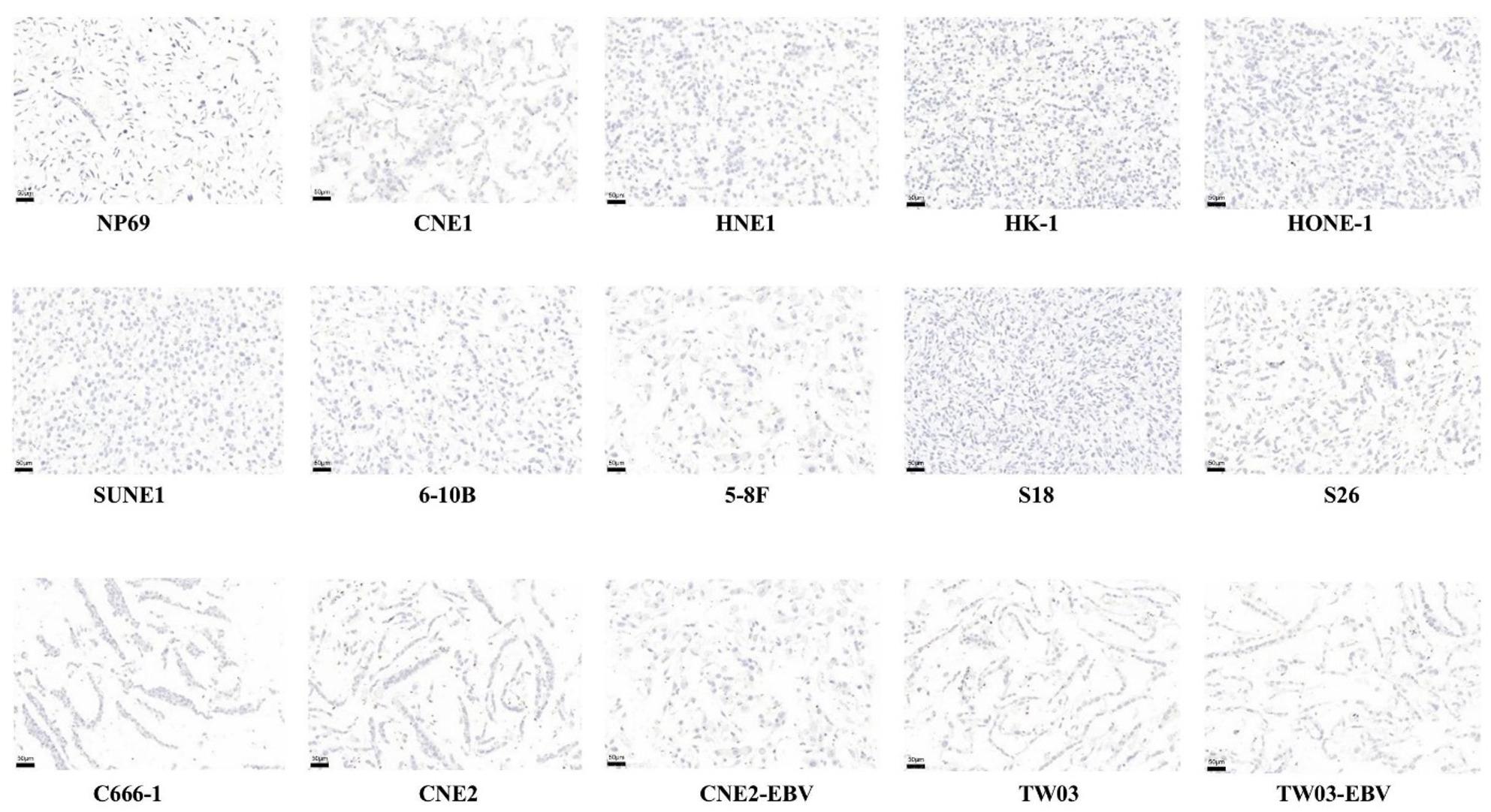


Correct Fig. 1: