# Supplementary figures and images for: Lymphocyte activating gene 3 protein expression in nasopharyngeal carcinoma is correlated with programmed cell death-1 and programmed cell death ligand-1, tumor-infiltrating lymphocytes
Source: Cancer Cell Int. 2021 Aug 28;21:458. doi: 10.1186/s12935-021-02162-w (PMC8403354; doi:10.1186/s12935-021-02162-w)

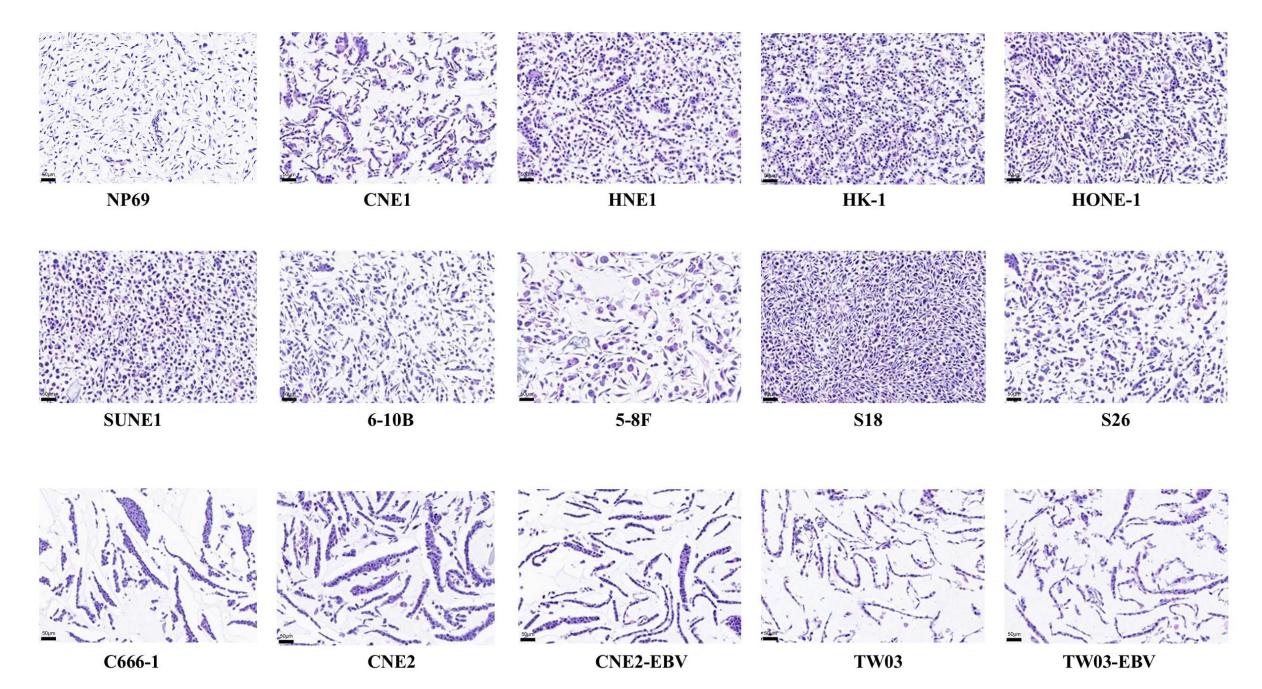


**Figure S1.** Hematoxylin-eosin (HE) staining of all 15 NPC cell lines (×20). Scale bars: 50μm.

Supplement: Supplementary file 1 — Additional file 1: Figure S1. Hematoxylin–eosin (HE) staining of all 15 NPC cell lines (× 20). Scale bars: 50 μm. [file 12935_2021_2162_MOESM1_ESM.docx]
